# Four Common Pesticides, Their Mixtures and a Formulation Solvent in the Hive Environment Have High Oral Toxicity to Honey Bee Larvae

**DOI:** 10.1371/journal.pone.0077547

**Published:** 2014-01-08

**Authors:** Wanyi Zhu, Daniel R. Schmehl, Christopher A. Mullin, James L. Frazier

**Affiliations:** 1 Department of Entomology, Center for Pollinator Research, The Pennsylvania State University, University Park, Pennsylvania, United States of America; 2 Honey Bee Research and Extension Laboratory, Department of Entomology and Nematology, University of Florida, Gainesville, Florida, United States of America; Goethe University Frankfurt, Germany

## Abstract

Recently, the widespread distribution of pesticides detected in the hive has raised serious concerns about pesticide exposure on honey bee (*Apis mellifera* L.) health. A larval rearing method was adapted to assess the chronic oral toxicity to honey bee larvae of the four most common pesticides detected in pollen and wax - fluvalinate, coumaphos, chlorothalonil, and chloropyrifos - tested alone and in all combinations. All pesticides at hive-residue levels triggered a significant increase in larval mortality compared to untreated larvae by over two fold, with a strong increase after 3 days of exposure. Among these four pesticides, honey bee larvae were most sensitive to chlorothalonil compared to adults. Synergistic toxicity was observed in the binary mixture of chlorothalonil with fluvalinate at the concentrations of 34 mg/L and 3 mg/L, respectively; whereas, when diluted by 10 fold, the interaction switched to antagonism. Chlorothalonil at 34 mg/L was also found to synergize the miticide coumaphos at 8 mg/L. The addition of coumaphos significantly reduced the toxicity of the fluvalinate and chlorothalonil mixture, the only significant non-additive effect in all tested ternary mixtures. We also tested the common ‘inert’ ingredient N-methyl-2-pyrrolidone at seven concentrations, and documented its high toxicity to larval bees. We have shown that chronic dietary exposure to a fungicide, pesticide mixtures, and a formulation solvent have the potential to impact honey bee populations, and warrants further investigation. We suggest that pesticide mixtures in pollen be evaluated by adding their toxicities together, until complete data on interactions can be accumulated.

## Introduction

Recently, one hundred and twenty one different pesticides and metabolites were identified in the hive with an average of seven pesticides per pollen sample, including miticides, insecticides, fungicides, herbicides, and insect growth regulators [1], [2]. Feeding on pollen and nectar in the larval diet directly exposes honey bee larvae transdermally, orally and internally [3]; therefore, the potential for chronic toxicity and synergistic interactions at the brood stage seems likely to occur, especially considering the fact that early life stages might be much more sensitive to certain contaminants relative to the adult stage. Several studies have demonstrated that insecticides ranging from insect growth regulators and encapsulated organophosphate formulations to systemic insecticides are more toxic to larvae than to adult bees [4]–[8]. Moreover, because beebread serves as an absolute requirement for developing bee larvae, pesticide disruption of the beneficial mycofloral community in the colony may thwart the processing of pollen into beebread and allow undesirable pathogens to thrive, therefore indirectly impacting the brood health [9], [10]. Indeed, chronic exposure to pesticides during the early life stage of honey bees may thus contribute to inadequate nutrition and/or direct poisoning with a resulting impact on the survival and development of bee brood [11]. Conceivably, these impacts on the larval phase could lead to weakening of the colony structure over time. To date, only a few peer-reviewed pesticide toxicity studies assess the risks of oral toxicity of pesticides to honey bee larvae. Therefore, a goal of our study was to assess the chronic and mixture effects of common pesticides at realistic exposure concentrations on larval honey bee survival. In order to mimic realistic exposure scenarios of honey bee larvae to contaminated pollen food, we chose the four most frequently detected pesticides in the hive - fluvalinate, coumaphos, chlorothalonil, and chlorpyrifos, and tested them alone and in all combinations via chronic dietary exposure, at concentrations found in pollen and beebread.

The pyrethroid *tau*-fluvalinate and the organophosphate coumaphos have been used widely for Varroa mite control, and found highly persistent in the hive with an estimated half-life in beeswax of about 5 years [12]. These compounds have shown evidence of synergistic toxicity on adult honey bees at the level of cytochrome P450-mediated detoxification [13]. Chlorothalonil, a broad-spectrum agricultural fungicide with an unclear mode of action [14], is often applied to crops in bloom when honey bees are present for pollination, because it is currently deemed safe to bees. However, some fungicides have shown direct toxicity to honey bees or solitary bees at field use rates [15] and fungicides in stored pollen are known to inhibit the growth of beneficial fungi thereby reducing the nutritional value of the pollen to bees [10]. Chlorpyrifos is a widely employed organophosphate in crop management [16] and its residues were frequently found in honey, propolis and dead bees. These in-hive (beekeeper applied) varroacides and out-of-hive (farmer applied) insecticides and fungicides may act alone or in concert, in ways currently unknown, to create a toxic environment for honey bee growth and development.

Another goal of this study was to examine the effect of an ‘inert’ ingredient on brood survival. Little data exist concerning the toxicity of ‘inert’ ingredients on honey bees, likely because bee toxicity information for pesticide formulations is not currently required by the U.S. Environmental Protection Agency as part of the pesticide registration process in contrast to the European Union where toxicity for representative formulations is mandatory [17]. Pesticide risk assessment is largely stymied by lack of public access to product-specific information of ‘inerts’ or co-formulants [18]. Some ‘inert’ ingredients such as those in formulations of the herbicide glyphosate are more toxic than active ingredients when tested on aquatic organisms [19]. That ‘inert’ more than active ingredients dominate pesticide formulations and spray tank adjuvants so to increase efficacy and stability of the pesticide makes it important to examine the role of ‘inerts’ on honey bee toxicity. Here, we studied the chronic toxicity of N-methyl-2-pyrrolidone (NMP, CAS 872-50-4) to bee brood development. The co-solvent NMP is used extensively in chemical processing and agricultural chemical formulations [20], [21]. The NMP tested alone or in formulations has demonstrated developmental toxicity in rats by various routes of administration [22] and also has shown high toxicity potential for aquatic invertebrates [23]. There is presently no information in the published literature regarding toxic effects of NMP to honey bees. Our study will be the first to test if this common ‘inert’ ingredient is toxic to honey bee larvae by continuous dietary exposure, and will serve as a foundation for future studies exploring ‘inert’ toxicity.

Specific objectives of the present study using the standardized *in vitro* larval feeding method developed by Aupinel et al. [24] are to: (i) assess possible toxic effects of single pesticides on the survival of individual *A. mellifera* larva during a 6-d continuous feeding with contaminated diet; (ii) compare the sensitivity difference between larval and adult bees to the same pesticide exposure; (iii) determine whether the selected pesticides in all combinations at realistic concentrations have any synergistic effects; and (iv) examine the toxicity of environmentally realistic levels of the formulation ingredient NMP on larval survival. Measurable impacts on larvae should demonstrate the need to extend pesticide risk assessment for honey bees from primarily acute effects on adults to chronic impacts on brood survival and development, and of the need to consider both active and ‘inert’ ingredients in formulations, so that more informed decisions can be made by governments, beekeepers and growers about pesticide application inside and outside the hive.

## Materials and Methods

### Acquisition of 1^st^ instar larvae

Honey bee (*A. mellifera*) 1^st^ instar larvae were collected from two colonies of *A. m. ligustica* strain reared in our experimental apiary (GPS Coordinates: 40°49′20″N, 77°51′33″W). In order to collect newly emerged larvae, a honey bee queen was confined in the queen excluder cage and placed in the 2^nd^ super from the bottom of the hive and positioned in the center of the super to allow for proper incubation of the newly laid eggs. After being caged for 30 h, the queen was released from the cage and eggs were incubated in the hive for 3.5 days. Frames of newly-hatched 1^st^instar larvae were taken to the laboratory in a pre-warmed chamber (∼35°C).

### Diet preparation

Honey bee larval diet (adaptation of [24]) was prepared using 50% royal jelly (Beenatura.com), 12% D-glucose (Fischer Chemical, Fair Lawn, NJ, USA), 12% D-fructose (Fischer Chemical, Fair Lawn, NJ, USA), 2% yeast extract (Bacto™, Sparks, MD, USA), and distilled water (24%). Royal jelly was preserved at −80°C until use. Ingredients minus royal jelly were completely dissolved and filtered through a 0.2 µm membrane (Corning) to remove particulate matter and bacteria. This solution was poured onto royal jelly that was free of wax particles, and mixed thoroughly at room temperature using a spatula. Diet was stored at 4°C for a maximum of three days prior to use.

### Pesticide application

The concentrations of applied pesticides were selected based on our previous laboratory findings of commonly found pesticides in pollen [1]. According to the survey of pesticide residues conducted on bee-related product samples from migratory and other beekeepers during the 2007–08 growing seasons, the most prevalent detections at 95th percentile values (levels at which only 5% of detections are higher) in trapped pollen samples were 0.3 mg/L (0.3 ppm) fluvalinate, 0.8 mg/L coumaphos, 0.15 mg/L chlorpyrifos, and 3.4 mg/L chlorothalonil (unpublished data up to 2009). Foraging bees may avoid and dilute contaminated pollen with that from alternative hosts; therefore, the level of contamination found in the trapped pollen pellets varies in relation to the foraging environment of the colony [1], [2], [25]. We have observed that apple pollen contributes approximately 10% of overall trapped pollen samples from hives placed in apple orchards during a 10-d pollination event (unpublished data). In addition, these pesticides have also been detected in other hive products at even higher levels including beebread, wax comb, foundation, and more rarely in bees. Developing bees are exposed to pesticide residues by contact with the wax, beebread and contaminated bees, so the level found in trapped pollen or royal jelly is not fully representative of actual exposure of larval bees to pesticides. For example, pollen residues of fluvalinate and coumaphos primarily originate by transfer from the contaminated comb wax, which contains much higher levels (e.g. 100-times) of these miticide residues [1], [2]. Therefore, in the absence of exact measures of pollen residues in larval foods, we chose to test at 10 times the levels of these four pesticides found in pollen samples. We mixed fluvalinate (purity, 95%), coumaphos (purity, 99%), chlorpyrifos (purity, 99%), and chlorothalonil (purity, 98%) purchased from Chem Service (West Chester, PA, USA) in the larval diet at nominal concentrations of 3, 8, 1.5, and 34 mg/L, respectively. Our calculated concentrations are in accordance with the maximal levels of pesticides detected in both trapped pollen and beebread samples and within the range of 95 percentile values of four selected pesticides detected in hive samples [1]. Therefore, we believe that applying a factor of 10 can give a rough but realistic estimation of the actual exposure of larval bees through contaminated diet or direct transfer from much higher residues in the comb.

Pesticide treatments included four pesticides tested alone and in two, three, and four-component mixtures. To prepare stock solutions, each technical grade pesticide was individually dissolved in acetone and methanol, respectively. Each test solution was mixed thoroughly into the artificial diet at specific concentrations and stored in 2 ml sterile glass vials (Corning, USA). We monitored three control groups in the study: untreated diet, one solvent-treated diet containing 1% methanol and another solvent control containing 1% acetone. We also tested the dietary toxicity of a range of N-methyl-2-pyrrolidone concentrations on larval survival. NMP can be used to 100% of the solvent in pesticide formulations [26]. Table S1 lists the percentage of the solvent NMP in some pesticide formulations that disclose it in MSDS. Here, we tested seven nominal concentrations including 0.01% (100 mg/L in diet), 0.02%, 0.05%, 0.1%, 0.2%, 0.5% and 1% (10,000 mg/L).

Each experiment was repeated twice including control (3 groups), single (6 treatment groups), mixture (binary mixtures: 6 treatment groups; ternary mixtures: 6 treatment groups; four-component mixtures: 2 treatment groups), and ‘inert’ toxicity tests (seven concentrations of NMP). Sample size for each treatment starting from the same experimental day is 3 replicates with 24 larvae per replicate.

### In vitro larval rearing technique

Newly hatched 1^st^ instar larvae were transferred from hive frames into sterile, 48-well culture plates (Corning, USA) for the *in vitro* rearing technique with 24 larvae per plate. Larval transfers were done in the lab without the use of a sterile hood. The sterile, push-in queen cups (B&B Honey Farm, USA) were placed in every other well. Diet was warmed to ∼34°C in a heating block prior to larval transfer. Using an Eppendorf 10–100 µl variable volume pipette, 10 µl of each diet treatment was placed per queen cup. A 00 camel hair paintbrush was used to transfer each larva from the cell on the frame to the cup. The paintbrush was dipped into distilled water between each larval transfer to aid in a smooth transfer, and was sanitized by dipping in 95% ethanol after every four to five transferred larvae. Larvae were placed directly on top of the diet and inspected for mobility to ensure a quality transfer. Four additional queen cups were equally spaced in four of the remaining open wells before placing the lid on the culture plate, allowing for adequate ventilation of the larvae throughout the experiment. Each plate was placed in a humidity chamber and kept at 95% relative humidity with a 10% aqueous solution of sulfuric acid being used at the base of the chamber to maintain humidity. Humidity chambers were placed in an incubator at 34°C in the dark and were not disturbed throughout the experiment, except when replacing the diet for ∼15 min/d.

For this study, only the survivorship of honey bees during the larval stage was monitored to evaluate the impacts of selected pesticides. Larval mortality was recorded daily by probing the larvae with sanitized forceps. The dead larvae were removed daily. Diet for each larval bee was replaced daily. Old diet was removed using a glass disposable pipette and new diet was immediately placed in each queen cup according to the following schedule to account for larval growth: day 1- 10 µl, day 2- 10 µl, day 3- 20 µl, day 4- 30 µl, day 5- 40 µl, and day 6- 50 µl.

### Kaplan-Meier survival analysis

The 6-d larval survival data were segregated by pesticide treatment and analyzed using Kaplan-Meier survival analysis [27]. This estimate generally assumes independence among the individual death events and randomization within the treatment group. The hazard rate h(*t*) is the conditional probability of failure or death in a small time period given that the subject has survived up until a specified time *t*. The greater the value of the hazard rate, the greater the probability of impending death. The null hypothesis of no difference between survival curves of treatment and control groups was tested by the Log-rank test that weights each death by the square root of the total number of individuals at risk per time interval, placing less emphasis on deaths occurring later in the experiment. All the survival analyses were implemented in SAS survival program (SAS/STAT® 9.2 User's Guide).

### Comparison between adult and larval sensitivity

The difference in sensitivity to the same pesticide between adult bees and larvae can be quantitatively evaluated by comparing the actual larval mortality per day from the *in vitro* test with the predicted mortality for adult bees if exposed to the same concentrations of pesticides. The larval mortality data were corrected with Abbott's formula beforehand. Here, the impacts of pesticide treatments on adult bees were estimated from the adult acute topical LD_50_ data converted to whole-bee LC_50_ values [1], because neither the chronic nor acute oral toxicity data of adult bees are currently available for all pesticides selected for this study. Predicted adult toxicity can be estimated as a function of the magnitude of toxicant exposure and the individual's sensitivity to a toxicant, which is generally characterized by the probit model [28].The predicted proportion of insects killed (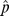), in probit transformed units, calculated as 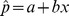 where *a* = intercept and *b* = slope from the regression of the transformed data and *x* is the log-transformed concentration or time. Results of probit analyses are reported typically as a concentration or time required to kill a certain proportion of the test insects (e.g., LC_50_). Table 1 shows the average LC_50_ values from the literature [1] and probit slopes from other sources [28]. One exception is chlorothalonil, which is estimated using the default probit slope of 4.5 because its mortality levels under topical or oral applications to honey bees are found to be insufficient to establish a dose-response relationship. Therefore, the probit function for each pesticide to adult honey bees can be inferred from the LC_50_ values (*x*), probit mortality (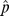 = 5) and probit slope (*b*) [13], [28]. Then, the probit model can be extrapolated to predict the probability of an impact of each pesticide on adult bee survival for a specified concentration. Using the Probit program in SAS 9.2 (SAS/STAT® 9.2 User's Guide), the predicted probit-type mortality can be transformed to the original percent units and compared with the actual larval percent mortality data. Using the compilation of acute data from different sources may complicate the accurate estimation of the adult toxicity because of the heterogeneity introduced by differences among the studies; however, given the limitations we felt this was a reasonable approach to obtain a first approximation of the differences in adult and larval sensitivity to the same pesticide exposure.

**Table 1:** Comparison between the predicted adult mortality rate (PM, %) for each tested concentration (Conc., mg/L) of four pesticides using a probabilistic toxicity model and the observed brood mortality rate (AOM, %) for bee larva from the 6-d *in-vitro* rearing experiments.

	Adult honey bee				Honey bee larva						
	Inverse probit prediction				*In-vitro* brood test						
Pesticide	β^a^	LC_50_ b	Conc.	PM^c^	1-d^d^	2-d^d^	3-d^d^	4-d^d^	5-d^d^	6-d^d^	AOM^e^
Fluvalinate	2.5	15.86	3	3.6	3.13*	8.06	12.28	10.00	11.11	68.85**	11.72
Coumaphos	2.9	46.3	8	1.4	6.25*	1.67	8.47	5.56	3.92	53.73**	8.60
Chlorothalonil	4.5	1110	34	4 E-10	0.00	8.93	7.84	12.77	7.32	56.60**	9.82
Chlorpyrifos	10	1.22	1.5	82	0.00	4.17	8.70	33.33**	32.14**	0.00	10.07

^a^β is the slope of the probit function for different pesticides [13], [28].

^b^LC_50_ is the median lethal concentrations of each pesticide to adult honeybees [1].

^c^PM = predicted adult mortality rate (%) for each pesticide at the tested concentrations using inverse prediction of the probit function.

^d^1,2,3,4,5,6-d is the observed conditional mortality rate (%) for larval bees at each age (in day) in the *in vitro* rearing process.

^e^AOM = average daily mortality rate (%) for larval bees in the *in vitro* rearing process.

^*^Significant at *p*<0.05;

^**^significant at *p*<0.001. (Statistical differences in larval survival were assessed between pesticide-treated and solvent control groups.)

### Pesticide interaction determination

We used significant departures from additive toxicity to define antagonistic and synergistic interactions between pesticides in mixtures [29]. The expected additive toxicity for the chemical mixture is the sum of each chemical's toxicity to larval survival, calculated as f chemical components in the pesticide mixture and *h_i_* is the hazard rate for a specific component estimated from the laboratory bioassay data. The sum of the responses (E*h_n_*) to the individual components is estimated based on the assumption that the selected pesticide mixtures are the combination of substances with independent modes of action or similar modes of action. The mixture toxicity can be predicted as follows: *Additive interactions–* Simultaneous action of components in which the observed response of honey bee larvae to a mixture (*h_n_*) is equal to the sum of the responses (E*h_n_*) to the individual components; *Synergistic interactions–*Simultaneous action of components in which *h_n_* is significantly higher than E*h_n_*; *Antagonistic interactions–*Simultaneous action of components in which *h_n_* is significantly less than E*h_n_*.

We did not test different concentrations of each pesticide component and of the combinations to fit dose-response curves. Neither food intake nor concentrations of pesticides consumed by each larva were measured during the oral feeding. Therefore, this method does not allow exact quantification of the level of interaction but makes only an initial qualitative assessment of synergism or antagonism.

## Results

### Control toxicity

**Figure 1:**
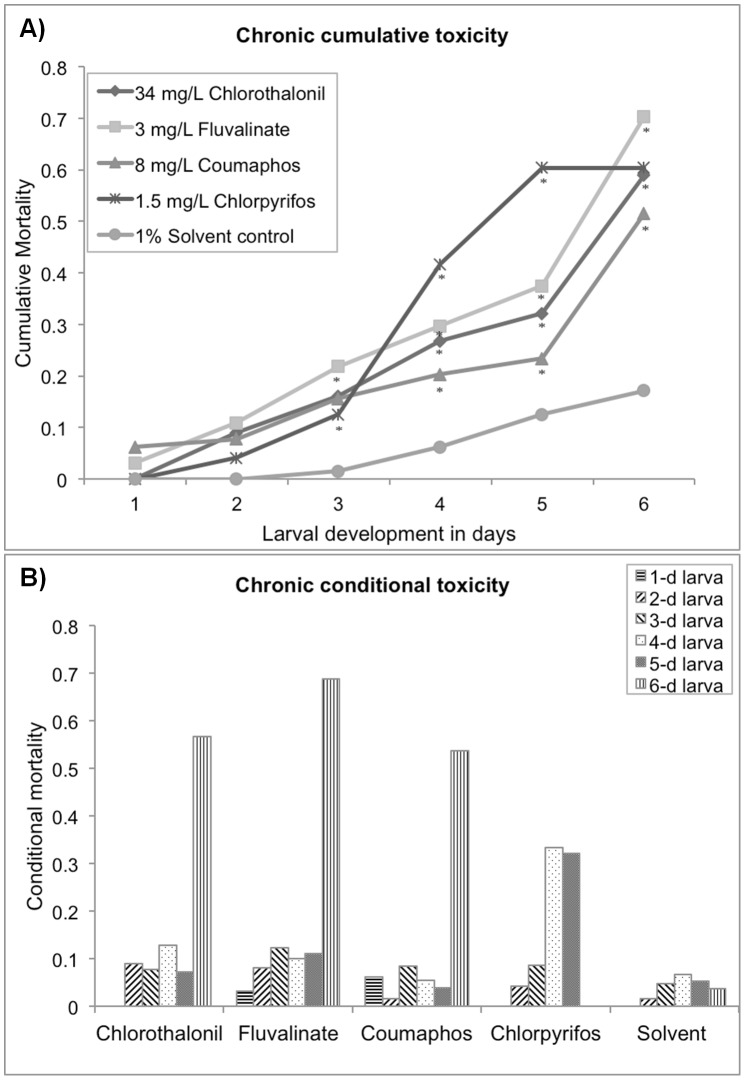
Larval survival during the 6-d development stage reared on artificial diet contaminated with four pesticides at the selected concentrations and a 1% solvent control. (A) shows the cumulative mortality of honey bee larvae through 6-d development continually exposed to 34 mg/L Chlorothalonil, 3 mg/L Fluvalinate, 8 mg/L Coumaphos, 1.5 mg/L Chlorpyrifos and 1% solvent; (B) illustrates the conditional mortality for different development stages of bee larva. Asterisks denote significant difference from the respective solvent controls (analysis of variance, Log-rank test, *p*<0.0001).

No significant differences in larval mortality were observed when larvae were reared on untreated artificial diet or diet mixed with 1% methanol or 1% acetone (Log-rank test, *p*>0.05) (data not shown). These three control groups showed an accumulative 6-d percent mortality of approximately 17.2% (Fig. 1), which is within the normal range observed for control mortality using the *in-vitro* larval rearing protocol [24], [30]. Because control mortality exceeds 10%, the larval mortality data from treatment groups were corrected with Abbott's formula.

### Single pesticide toxicity

Chronic exposure of bee larvae to each of the four pesticides at tested concentrations showed significant toxic effects on larval survival (Log-rank test, *p*<0.0001), resulting in an overall 2- to 4-fold reduction in the total 6-d percentage survival compared to the control mortality (Fig. 1A). Based on age-specific toxicity data, mortality rates for each pesticide were uneven across different larval stages (Fig. 1B). For 1-day-old larvae, 8 mg/L coumaphos and 3 mg/L fluvalinate were more toxic than the other two pesticides. The 2 and 3-day-old larvae showed similar sensitivity to different pesticide exposures, approximately 10% mortality per day. The 4 and 5-day-old larvae were most sensitive to 1.5 mg/L chlorpyrifos, causing more than 32% larval death each day (Table 1). A dramatic increase in larval mortality for 6–day-old larvae was observed in 34 mg/L chlorothalonil and the two miticide groups, ranging from 53.73% to 68.85%. Using the probit model, notable differences were found in pesticide sensitivity between the adult bee and larvae (Table 1). Among the four pesticides tested, 1.5 mg/L chlorpyrifos was the only treatment that adult bees were more susceptible to than the larvae. For the other pesticides, the larvae showed increased sensitivity over that of adult bees. Notably, chlorothalonil at the sublethal concentration of 34 mg/L was least toxic to adult bees, however most toxic to larvae followed by 8 mg/L coumaphos and 3 mg/L fluvalinate. On average, coumaphos was the least toxic to larval bees among the four pesticides.

### Synergistic interactions

#### I. Chronic toxicity of chlorothalonil and coumaphos

**Figure 2:**
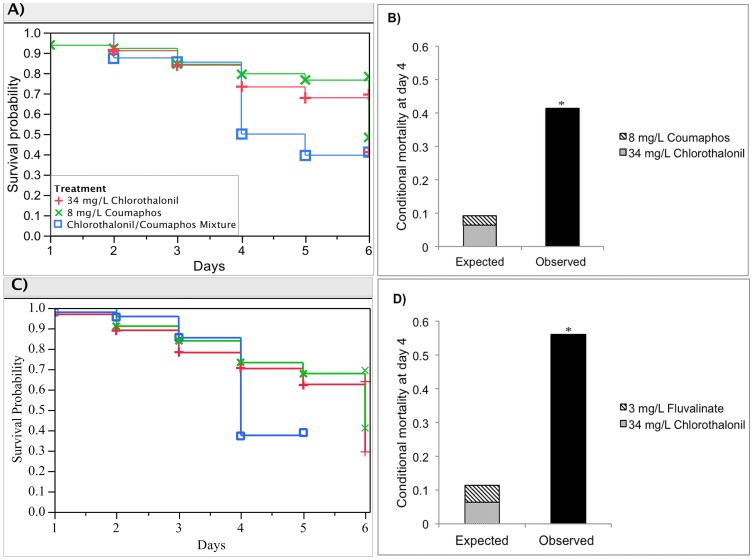
Synergistic interactions for two pairs of pesticide mixtures: 8 mg/L Coumaphos, 34 mg/L Chlorothalonil and the mixture; 3 mg/L Fluvalinate, 34 mg/L Chlorothalonil and the mixture. (A) and (C) show the respective Kaplan-Meier survival plots for honey bee larvae reared for each pair of pesticide mixture; (B) and (D) illustrate the interaction determination based on the deviation of observed mixture toxicity (black bar) from the expected additive toxicity (stacked bar). Asterisks denote significant difference from the expected additive toxicity (Mann–Whitney test, *p*<0.0001).

The effects of chlorothalonil (34 mg/L), coumaphos (8 mg/L), and their mixture on larval survival through the 6-d development are shown in Fig. 2A. In the first 3 days of larval rearing, these three groups exhibited similar survival curves (p = 0.1988, Log-rank test). Subsequently, the larvae reared on the diet contaminated with the chlorothalonil/coumaphos mixture died most quickly. The risk of 4-day-old larvae being killed by the mixture was higher than for the other stages of larvae and the single pesticide groups. The hazard rate of the combination group (hn(4) = 0.523) was 9-times higher than the coumaphos group (hCM(4) = 0.057) and 3-times higher than the chlorothalonil group (hCL(4) = 0.136). The conditional probability of 4-day-old larvae being killed by the mixture treatment was 5-times higher than that of expected additive toxicity (Fig. 2B, Ehn(4) = 0.0965, p<0.0001, Mann–Whitney test). Therefore, the pairing of chlorothalonil and coumaphos produced a significant synergism on mortality of larvae older than 4 days.

#### II. Chronic toxicity of chlorothalonil and fluvalinate

For the 4-day-old larvae, the hazard rate of the mixture (hn(4) = 0.78) was the highest during the 6-d larval development, which was 7-times higher than the fluvalinate (3 mg/L) group (hFlu(4) = 0.105) and 5-times higher than the chlorothalonil (34 mg/L) group (hCL(4) = 0.136) (Fig. 2C). The chlorothalonil/fluvalinate mixture at the tested concentrations gave a synergistic interaction, which significantly magnified the hazard rate by 7 fold over the sum of the individual effects (Fig. 2D, Ehn(4) = 0.121, p<0.0001, Mann–Whitney test).

### Additive interactions

#### I. Chronic toxicity of fluvalinate and chlorpyrifos

**Figure 3:**
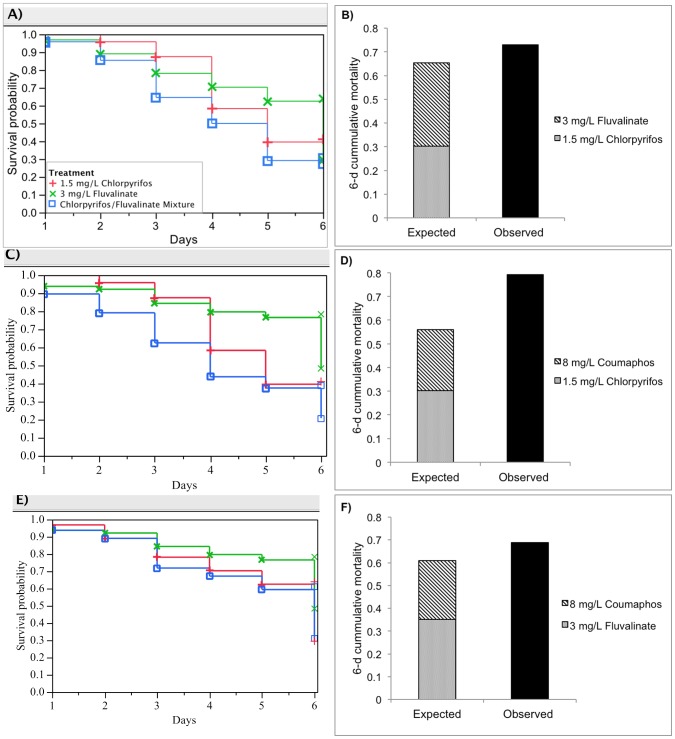
Additive effects for three pairs of pesticide mixtures: 3 mg/L Fluvalinate, 1.5 mg/L Chlorpyrifos and the mixture; 8 mg/L Coumaphos, 1.5 mg/L Chlorpyrifos and the mixture; 8 mg/L Coumaphos, 3 mg/L Fluvalinate and the mixture. (A), (C) and (E) show the respective Kaplan-Meier survival plots for honey bee larvae reared for each pair of pesticide mixture; (B), (D) and (F) illustrate the interaction determination based on the deviation of observed mixture toxicity (black bar) from the expected additive toxicity (stacked bar).

Larval survival on fluvalinate (3 mg/L) and chlorpyrifos (1.5 mg/L) declined the fastest among pesticide mixture treatments, ranging from 4.17% to 70.83% (Fig. 3). No significant differences were found in larval survival between single component groups through the 6-d development (Fig. 3A, Log-rank test, p = 0.1711). This binary combination produced additive toxicity. The 6-d cumulative percent mortality caused by this mixture (hn = 71%) was slightly higher than the sum of the response to single components, but not at a significant level (Fig. 3B, Ehn = 48.96%, p = 0.171, Mann–Whitney test).

#### II. Chronic toxicity of chlorpyrifos and coumaphos

The larval chronic toxicity of this combination treatment was the highest among tested pesticide mixtures causing from 10.4% to 79.2% mortality during the 6 days. Survival was least affected by the diet with 8 mg/L coumaphos (Fig. 3C). The interaction between these pesticides showed an additive effect. The 6-d cumulative percent mortality of larvae reared on the mixture (hn = 79.2%) did not differ significantly from expected additive toxicity (Fig. 3D, Ehn = 56%, p = 0.558, Mann–Whitney test).

#### III. Chronic toxicity of fluvalinate and coumaphos

The survivorship of larval bees on the combination and fluvalinate alone treatments exhibited a similar gradual declining trend, achieving the highest cumulative mortality at the end of the 6-d development (Fig. 3E). Both showed more toxicity to larval bees than coumaphos alone (Fig. 3E, p = 0.0425, Log-rank test). Fluvalinate and coumaphos, mixed at 3 mg/L and 8 mg/L respectively, showed an additive effect. The accumulative percent mortality in the mixture group (hn = 68.75%) did not vary significantly from the expected additive toxicity (Fig. 3F, Ehn = 60.94%, p = 0.052, Mann–Whitney test).

### Antagonistic interactions

#### I. Chronic toxicity of fluvalinate and chlorothalonil at low concentrations

**Figure 4:**
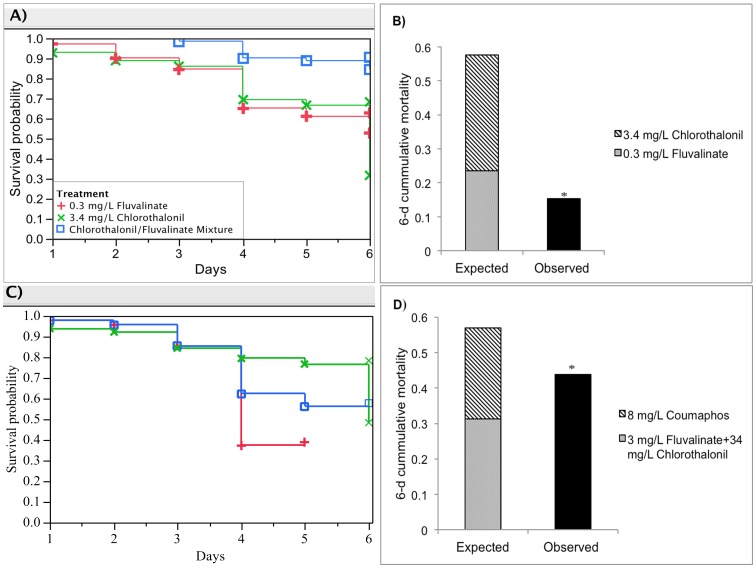
Antagonistic interactions for two pairs of pesticide mixtures: 0.3 mg/L Fluvalinate, 3.4 mg/L Chlorothalonil and the mixture; 3 mg/L Fluvalinate+34 mg/L Chlorothalonil mixture, 8 mg/L Coumaphos and the three-component mixture. (A) and (C) show the respective Kaplan-Meier survival plots for honey bee larvae reared for each pair of pesticide mixture; (B) and (D) illustrate the interaction determination based on the deviation of observed mixture toxicity (black bar) from the expected additive toxicity (stacked bar). Asterisks denote significant difference from the expected additive toxicity (Mann–Whitney test, *p*<0.0001).

The 3.4 mg/L chlorothalonil and 0.3 mg/L fluvalinate mixture showed the least toxicity to larval development among pesticide combinations tested (Fig. 4A). Especially, for the 4-day-old larva, the hazard rate of individual component groups (hCL(4) = 0.214, hFlu(4) = 0.259) was greater than twice the mixture treatment (hn(4) = 0.088). This mixture showed antagonistic interaction, significantly reducing the hazard rate of 4-day-old larvae by three-fold from the expected additive toxicity (Fig. 4B, Ehn(4) = 0.2365, p<0.0001, Mann-Whitney Test).

### Three-component mixture toxicity

All six possible pairings were selected to determine the toxicity for three-component mixtures including chlorothalonil/fluvalinate/coumaphos and fluvalinate/coumaphos/chlorpyrifos. The only significant difference found was when coumaphos (8 mg/L) was added to the two-component mixture of fluvalinate (3 mg/L) and chlorothalonil (34 mg/L), giving a 3% reduction in the 6-d accumulative larval mortality (*h_n_* = 38%) from the expected additive effect (Fig. 4C and 4D; E*h_n_* = 41.41%, *p* = 0.006, Mann-Whitney Test). The other five pairings did not yield significant changes in larval survival when adding one component into the existing binary mixtures.

### Four-component mixture toxicity

Two pairings of mixtures including chlorothalonil added to fluvalinate/coumaphos/chlorpyrifos and chlorpyrifos added to chlorothalonil/fluvalinate/coumaphos were tested at the same concentrations as before to determine toxicity interactions in going from three- to four-component mixtures. There were no significant changes in larval survival when integrating a fourth component into these three-component mixtures. The four-component mixture caused 54.17% larval mortality at the end of the 6-d larval development.

### ‘Inert’ ingredient toxicity

**Figure 5:**
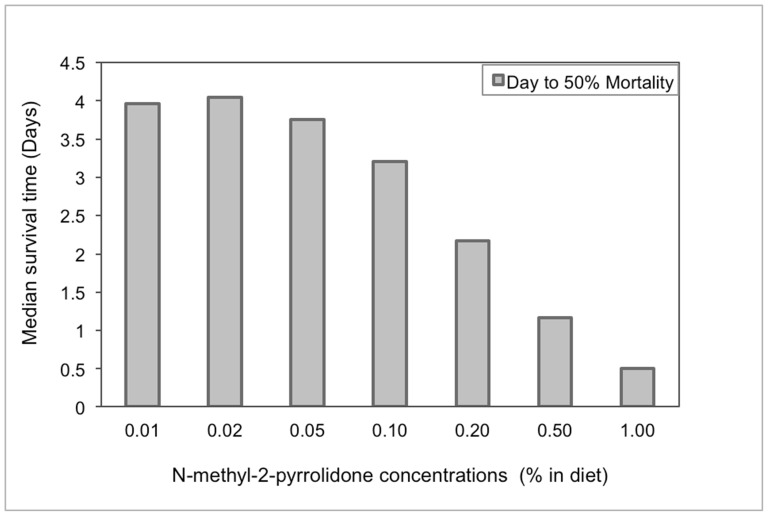
The estimated time to cause 50% larval mortality by seven nominal concentrations of N-methyl-2-pyrrolidone mixed in larval diet.

Chronic exposure of bee larvae to the ‘inert’ ingredient NMP at seven different concentrations ranging from 0.01% to 1% greatly impacted larval survival (Fig. 5). Increasing amounts of NMP correspondingly increased larval mortality. A 1% concentration (10,000 mg/L) of NMP was the most acutely toxic, generating 100% mortality within 24 h after treatment. Even for the lowest concentration of 0.01% (100 mg/L), the estimated time to cause 50% larval mortality was 4 days.

## Discussion

### Chronic toxicity

Our findings suggest that chronic dietary feeding at hive levels of common pesticide ingredients including the fungicide chlorothalonil, miticides fluvalinate and coumaphos, and insecticide chloropyrifos, individually or in mixtures, have statistically significant impacts on honey bee larval survivorship. A significant increase in larval mortality was found at or beyond 4-d of feeding. This is the first study to report serious toxic effects on developing honey bee larvae of dietary pesticides at measured hive residue concentrations. The maximum concentrations of fluvalinate, coumaphos, chlorothalonil, and chlorpyrifos found in our hive samples are 204 mg/L, 94.1 mg/L, 98.9 mg/L, and 0.9 mg/L, respectively (Table S2), which are much higher for the miticides and fungicide, or similar for the insecticide, to those levels tested here (Table 1). This chronic (6-d) toxicity is likely to be undetected in a conventional acute (24/48 h) toxicity study, resulting in potential underestimation of pesticidal effects. The lethal effects on honey bee larvae appearing after 4-d continuous exposure to pesticides at low concentrations are also observed in adult honey bees. The accumulated dose of the organophosphorus insecticides acephate, methamidophos or dimethoate resulting in 50% adult bee mortality was over 100-fold lower than the respective acute 24 h oral LD_50_ [31]. For these organophosphates and also the pyrethroids tested, their toxicity to worker bees was significantly increased by continuous versus single ingestion of the contaminated food. At low doses of imidacloprid, adult bee mortality was observed only 72 h after onset of feeding in contrast to immediate effects at much higher doses [32].

The causes for chronic larval bee toxicity for 6-d dietary subacute pesticide exposures remain unknown. It may be associated with the extended time needed to accumulate sufficient insecticide concentrations internally to exert nerve action at central target sites, which is consistent with the pharmacological receptor theory; or may reflect variation in honey bee detoxification capacities from the more peripheral to internal tissue sites. For instance, the results of high toxicity of low doses of all imidacloprid metabolites suggest the existence of binding sites with different affinities in honey bees [32]. Another explanation may be that honey bee detoxification mechanisms are not induced by chronic exposure of low concentrations of active substances, but require higher more acute concentrations to impact honey bee susceptibilities. In the former case, bee mortality would be latent due to the time needed for pesticide bioaccumulation, further favored by the more lipophilic pesticides fluvalinate, coumaphos, chlorpyrifos and chlorothalonil tested here. The latter case of acute higher concentrations driving induction of detoxification enzymes can result in both antagonistic and synergistic effects on the target-effective insecticide concentration depending on if the induced cytochrome P450 first activates (e.g., chlorpyrifos, coumaphos to respective oxons) or detoxifies (e.g., fluvalinate) the insecticide [33], [34]. Other induced enzymes (e.g., hydrolases, glutathione transferases) will further degrade and detoxify the primary metabolites.

It is also plausible that more general stress mechanisms (e.g., altered feeding, suppressed growth) dominate the chronic response. For example, exposures of some repellent pesticides such as pyrethroids at sublethal levels have been demonstrated to impair feeding behaviors of honey bees and bumble bees [3], [8]. In the case of honey bee larvae, they retain internally all metabolic wastes throughout the larval stage up to the pupal molt after which they defecate a waste pellet called the meconium [25]. Concentrations of pesticides and metabolites within brood tissues may result in continuous pesticide stress [35], which differs from the adult honey bee and most other insects where excretion of toxic wastes regularly occurs. Little information is available on the distribution of fluvalinate [36] and coumaphos [37] and their degradates in honey bee adults and brood. Further studies to examine the distribution and accumulation of fluvalinate, coumaphos, chlorpyrifos and chlorothalonil and their metabolites, in honey bees at different developmental stages are needed. Meanwhile, how honey bees at different life stages withstand chronic exposure need more detailed study of metabolic regulation in this social insect.

Remarkably, among the four pesticides tested in the present study, immature honey bees are highly vulnerable to the common fungicide chlorothalonil (Figs. 1 and 2). Dietary chlorothalonil killed more than 50% of larvae in 6 days at a level of 34 mg/L, a nontoxic dose to adult bees in acute bioassays (Table 1). This difference in larval to adult susceptibility was the largest among the four pesticides tested. It is unclear why, larval bees exhibited much greater sensitivity to chlorothalonil compared to adult bees; however, the present results demonstrate that investigating fungicide impacts on honey bees is particularly necessary for a realistic evaluation of pesticide impacts on colony health, given the frequent detections of chlorothalonil in pollen and wax samples. Hence, considering that honey bees are experiencing a diverse array of agrochemicals in the hive, the chronic toxicity test may better assess pesticide exposure for a honey bee colony.

### Mixture toxicity

Currently, studies of mixture toxicity between different classes of pesticides at concentrations of environmental relevance are rarely available for honey bees [34]. The present study of four pesticides in all combinations is the first study to investigate the potential synergism of common pesticides at realistic exposure levels to larval bees. The present results showed interactions between binary combinations of synthetic pesticides tested were mostly additive, which can be attributed to the same or independent mode of actions of the pesticides involved [33], [34]. For instance, additivity of the coumaphos/chloropyrifos mixture may be explained by their identical action as organophosphate inhibitors of acetylcholinesterase. The additive toxicity of the pyrethroid fluvalinate with either coumaphos or chloropyrifos is probably due to the independent primary action of the former on nerve sodium channels. Our result with larvae is not consistent with the adult honey bee study of Johnson et al., where the combination of fluvalinate and coumaphos was synergistic [13]. This discrepancy may be explained by the different life stage, lower insecticide concentration levels, and longer length of exposure used here.

The three and four component mixtures of tested pesticides have mostly demonstrated additive effects in larval bees. This finding is in general agreement with the Funnel Hypothesis [38], which states that the toxicity will tend towards concentration additivity as the number of components in equitoxic mixtures increases. One exception was the significantly less than additive response when coumaphos was integrated into the fluvalinate and chlorothalonil mixture. That coumaphos antagonizes the synergistic effect of fluvalinate and chlorothalonil may be related to its possible induction of the detoxification of one or both of the other pesticides. This anomaly may be related to the observation that elevated coumaphos levels in brood had the highest discriminatory value with regard to healthy bee colonies whereas higher levels of this miticide in the pollen food correlated with colony collapse [39], again indicating that pesticide susceptibilities differ across honey bee developmental stages.

Remarkably, binary mixtures of chlorothalonil with the miticides fluvalinate or coumaphos were synergistically toxic to 4-day-old bee larvae. This is the first demonstration for honey bee brood of a synergistic interaction between dominant in-hive miticides and the frequently-encountered fungicide chlorothalonil at environmentally relevant concentrations. Synergism with chlorothalonil and fluvalinate but not coumaphos for adult honey bee toxicity has been noted previously [40], [41].

Surprisingly, a significant antagonism was found for larval toxicity from the fluvalinate-chlorothalonil combination at one-tenth of the concentrations (Fig. 4) that otherwise exhibited a five-fold synergism (Fig. 2). One rationale behind this latter interaction, beyond the fact that the very diverging pyrethroid-multi-site chlorothalonil mechanisms of action may alone elicit synergistic effects, is that the high concentrations may directly inhibit detoxification enzymes. For example, the competitive inhibition of cytochrome P450 monooxygenase enzymes has been suggested to explain the synergistic interactions among pesticides for adult honey bees such as pyrethroid insecticides or mixtures of organophosphate insecticides and ergosterol biosynthesis inhibiting fungicides [42], [43]. Also, synergism between chlorothalonil and the herbicide atrazine has been documented in aquatic species [44]. Modes of action for chlorothalonil range from inhibiting glutathione and other thiol-dependent enzymes or protein receptors, to disrupting or degrading cell membranes causing lysis that can enhance penetration of other pesticides [14]. The tendency toward antagonism of brood toxicity at the lower dietary chlorothalonil-fluvalinate concentration may be associated with alternative peripheral mechanisms such as gut microbial detoxification that may be overwhelmed at higher dosage where more internal neurotoxic effects of the pyrethroid can prevail. The consequence is that biphasic low and high dose response relationships may result depending on the extent of multiple peripheral and internal sites of action that diverge in sensitivity to the toxicants as well as to the available detoxification pathways that differ in a tissue-dependent manner to the concentrations required for their induction.

While the mechanisms of interactions among pesticides with diverse modes of action and their dynamics in the developing honey bee larvae are not known, application of the concentration-addition model combined with chronic feeding tests represents a starting point for investigation of mixture effects at realistic levels and their risks for this pollinator. Considering that the diverse arrays of chemicals [1], [2], [45] and general additivity exist in the hive environment, examining the toxicity of chemical mixtures in addition to single toxicants is critical for a realistic assessment of pesticide hazards experienced by honey bees and other non-target organisms. In today's agriculture dominated by mass monocultures, adults and larvae of *A. mellifera* are inevitably exposed to transgenic material via pollen consumption of GM-crops [46], which might be another confounding factor for bee health. Although minor evidence showed adverse effects of Bt-crops on *A. mellifera*, the risk assessment of combined effects of Bt-crops and pesticides are completely lacking [47]–[49]. Hence, the dose dependency of the synergy, the multitude of compounds, the differences in adult bees and larvae, the possibility of continuous exposures, and the interaction with GM pollen should be taken into account in the environmental risk assessment.

### ‘Inert’ toxicity

Another important health issue that involves pesticide formulations and bees is the consequence of the additives or so-called non-active ingredients. The commonly-used ‘inert’ solvent N-methyl-2-pyrrolidone was found here to be highly toxic to larval honey bees (Fig. 5). Unfortunately, despite the potential toxicity of ‘inert’ ingredients and their widespread use in pesticide products, their testing and risk assessment seems to be inadequate. There is a growing body of research that has reported a wide range of adverse effects of ‘inert’ ingredients to human health, including enhancing pesticide toxicities across the nervous, cardio-vascular, respiratory, and hormonal systems [18], [50], [51]. However, limited data exist on the potential impacts of ‘inerts’ on non-target pollinators, although recent studies implicate formulation additives or adjuvants as key risk factors [52]. As one example, the toxicity of the fungicide captan to honey bee brood development was attributed to formulation ingredients other than the active ingredient alone [53]. The lack of detailed information of the usage of formulation ingredients greatly impedes appropriate risk assessment of ‘inert’ ingredient toxicity; therefore, label disclosure of the composition of pesticide formulations would facilitate this much-needed evaluation.

## Conclusions

The current study demonstrates the chronic oral and mixture toxicity of common pesticides at hive levels to honey bees at the larval stage. Most notable are the chronic larval toxicities of the fungicide chlorothalonil and its synergistic combinations with frequently used in-hive miticides, and the unexpected high toxicity of the formulation ingredient N-methyl-2-pyrrolidone. Considering the extensive detection of chlorothalonil and its coexistence with other pesticides in diverse combinations especially in hive pollen and wax, and its substantial larval toxicity alone and in mixtures shown here, the application of this and other fungicides during crop bloom cannot be presumed innocuous to pollinating honey bees. Given the critical sensitivity of larvae to chlorothalonil and its complex interactions with other pesticides, the potential impacts of fungicides on colony survival and development need further investigation. In the more complex milieu of this social insect and its aging hive environment, pesticides, formulation additives and their resulting mixtures may have greater long-term impacts on colony health than previously considered. Consequently, the scope of pesticide risk assessment for non-target honey bees should be expanded from the present emphasis on acute toxicity of individual pesticides to a priority for assessment of chronic and mixture toxicities that incorporate fungicides, other pesticide pollutants and their ‘inert’ ingredients.

## Supporting Information

Table S1 **Some pesticide formulations that disclose in msds the percentage of the solvent NMP.**
(DOCX)

Table S2 **Pesticide detections in 329 wax and 496 pollen samples collected 2007–12 from North American honey bee colonies.**
(DOCX)
