# Supplementary material for: Four Common Pesticides, Their Mixtures and a Formulation Solvent in the Hive Environment Have High Oral Toxicity to Honey Bee Larvae
Source: PLoS One. 2014 Jan 8;9(1):e77547. doi: 10.1371/journal.pone.0077547 (PMC3885384; doi:10.1371/journal.pone.0077547)
Supplement: Table S1 — Some pesticide formulations that disclose in msds the percentage of the solvent NMP. (DOCX) [file pone.0077547.s001.docx]

**Table S1.** Some pesticide formulations that disclose in msds the percentage of the solvent NMP.

| **Formulation^#^** | **Registrant** | **Active Ingredient(s)** | **% NMP** ^†^ |
| --- | --- | --- | --- |
| Abamectin E-AG^®^ 0.15EC | Etigra | abamectin | ≤ 30 |
| Acatak^®^ Pour-On Tick Inhibitor | Novartis | fluazuron | 30-60 |
| Advantix^®^ for Dogs | Bayer | imidacloprid, permethrin | 30-60 |
| Agri-Mek^®^ or Ardent^®^ 0.15EC | Syngenta | abamectin | ≤ 30 |
| Alto^®^ 100 SL Fungicide | Syngenta | cyproconazole | ≤ 10 |
| Assert^®^ Herbicide | Nufarm | imazamethabenz methyl ester | 49 |
| Assure^®^ II Herbicide | DuPont | quizalofop-p-ethyl | < 8 |
| Baycor^®^ 300, Turf Fungicide | Bayer | bitertanol | 54 |
| Bayfidan^®^ 250 EC Fungicide | Bayer | triadimenol | 60 |
| Campaign^®^ Herbicide | Monsanto | isopropylamine salts of glyphosate, 2,4-D | ≤ 7 |
| CFT Legumine™ Piscicide | CWE Properties | rotenone | 10 |
| Confidor^®^ SL 200 | Bayer | imidacloprid | 38 |
| Confidor^®^ Supra | Bayer | imidacloprid, cyfluthrin | 87 |
| Crown^®^ 225SL | Scotts | acetamiprid | 30 |
| Diamond^®^ 0.83EC | Makhteshim Agan | novaluron | 40-50 |
| Goal^®^ 2XL EC Herbicide | Dow | oxyflurofen | 10 |
| Jaguar^®^ Selective Herbicide | Bayer | bromoxynil octanoate diflufenican | 15 |
| Ortho^®^ Systemic Insect Killer | Ortho | acephate | < 25 |
| Quizalofop 200 EC Herbicide | 4Farmers | quizalofop-p-ethyl | 15 |
| Ricestar^®^ Herbicide | Bayer | fenoxaprop-p-ethyl, isoxadifen-ethyl | 15 |
| Saprol^®^ Fungicide | BASF | triforine | 24 |
| Score^®^ Foliar Fungicide | Syngenta | difenoconazole | 10-30 |
| Sphere^®^ Fungicide | Bayer | cyproconazole, trifloxystrobin | 55 |
| Temprano^®^ | Chemtura | abamectin | 15-40 |
| Tilt Xtra^®^ Fungicide | Syngenta | cyproconazole, propiconazole | < 20 |
| Zoro^®^ | Cheminova | abamectin | 15-40 |

**^#^**Insecticide or miticide unless noted otherwise.

**^†^**N-methyl-2-pyrrolidone; application rate of pesticides range from 0.05 to 100% of formulation diluted in water with an 1% average per product, and rates increasing from insecticide > fungicide > herbicide.
