# Supplementary material for: Four Common Pesticides, Their Mixtures and a Formulation Solvent in the Hive Environment Have High Oral Toxicity to Honey Bee Larvae
Source: PLoS One. 2014 Jan 8;9(1):e77547. doi: 10.1371/journal.pone.0077547 (PMC3885384; doi:10.1371/journal.pone.0077547)
Supplement: Table S2 — Pesticide detections in 329 wax and 496 pollen samples collected 2007–12 from North American honey bee colonies. (DOCX) [file pone.0077547.s002.docx]

**Table S2.** Pesticide detections in 329 wax and 496 pollen samples collected 2007-12 from North American honey bee colonies.

| **Total Pesticide*** | **CLASS^#^** | **Wax Samples with Detections (ppb)** | | | | | | **Pollen Samples with Detections (ppb)** | | | | | | **LOD^¶^** |
| --- | --- | --- | --- | --- | --- | --- | --- | --- | --- | --- | --- | --- | --- | --- |
|  |  | **%** | **Low** | **High** | **Mean** | **SEM^§^** | **95%tile** | **%** | **Low** | **High** | **Mean** | **SEM^§^** | **95%tile** |  |
| Fluvalinate | PYR | 93 | 2 | 204000 | 6823 | 801 | 27440 | 72 | 1 | 2670 | 108 | 13 | 331 | 1 |
| Coumaphos | OP | 95 | 1 | 94131 | 3042 | 414 | 10847 | 59 | 1 | 5828 | 176 | 18 | 826 | 1 |
| Chlorpyrifos | OP | 58 | 1 | 890 | 17 | 4 | 37 | 44 | 0.1 | 830 | 42 | 4 | 189 | 0.1 |
| Chlorothalonil | FUNG | 42 | 1 | 53700 | 985 | 271 | 2024 | 50 | 1 | 98900 | 2318 | 439 | 15832 | 1 |
| 1-Methylpyrrolidinone (NMP) | INERT | 9 | 1690 | 5420 | 3555 | 563 | 5234 | 0 | --- | --- | --- | --- | --- | 500 |

| *****Coumaphos includes degradates coumaphos oxon, chlorferone and potasan. |
| --- |
| **^#^**Class: FUNG = fungicide, INERT = formulation ingredient; OP = organophosphate, PYR = pyrethroid. |
| **^¶^**LOD = limit of detection (ppb). |
| **^§^**Mean and SEM for detections > LOD. |
